# Impact of Structural Compliance of a Six Degree of Freedom Joint Simulator on Virtual Ligament Force Calculation in Total Knee Endoprosthesis Testing

**DOI:** 10.3390/life14040531

**Published:** 2024-04-21

**Authors:** Eric Kleist, Paul Henke, Leo Ruehrmund, Maeruan Kebbach, Rainer Bader, Christoph Woernle

**Affiliations:** 1 Chair of Technical Mechanics/Dynamics, Faculty of Mechanical Engineering and Marine Technologies, University of Rostock, Justus-von-Liebig-Weg 6, 18059 Rostock, Germany; woernle@uni-rostock.de; 2 Biomechanics and Implant Technology Research Laboratory, Department of Orthopedics, Rostock University Medical Center, Doberaner Straße 142, 18057 Rostock, Germany; paul.henke@med.uni-rostock.de (P.H.); leo.ruehrmund@med.uni-rostock.de (L.R.); maeruan.kebbach@med.uni-rostock.de (M.K.);

**Keywords:** biomechanics, joint simulator, structural compliance, knee joint dynamics, total knee endoprostheses, multibody model

## Abstract

The AMTI VIVO™ six degree of freedom joint simulator allows reproducible preclinical testing of joint endoprostheses under specific kinematic and loading conditions. When testing total knee endoprosthesis, the articulating femoral and tibial components are each mounted on an actuator with two and four degrees of freedom, respectively. To approximate realistic physiological conditions with respect to soft tissues, the joint simulator features an integrated virtual ligament model that calculates the restoring forces of the ligament apparatus to be applied by the actuators. During joint motion, the locations of the ligament insertion points are calculated depending on both actuators’ coordinates. In the present study, we demonstrate that unintended elastic deformations of the actuators due to the specifically high contact forces in the artificial knee joint have a considerable impact on the calculated ligament forces. This study aims to investigate the effect of this structural compliance on experimental results. While the built-in algorithm for calculating the ligament forces cannot be altered by the user, a reduction of the ligament force deviations due to the elastic deformations could be achieved by preloading the articulating implant components in the reference configuration. As a proof of concept, a knee flexion motion with varying ligament conditions was simulated on the VIVO simulator and compared to data derived from a musculoskeletal multibody model of a total knee endoprosthesis.

## 1. Introduction

The human knee joint is an injury-sensitive joint in the human body [1]. It is a complex structure that plays a critical role in supporting activities of daily living. In this context, understanding knee joint biomechanics is essential for diagnosing and treating various musculoskeletal disorders. Kinematically, the knee joint is part of the lower extremity as an open kinematic chain. It can be modeled, e.g., as a revolute joint when only considering motions in the sagittal plane [2] or as a joint allowing translations and rotations in all spatial directions [3]. Muscles and ligaments act as active and passive force elements between the bony structures of the lower extremity. To unravel the intricate interplay of forces, kinematics, and soft tissue responses within the knee joint, physical joint simulators have proven to be a valuable tool for biomechanical investigations [3,4,5,6,7,8,9,10,11,12,13,14,15]. They allow preclinical testing of joint endoprostheses and human specimens under realistic conditions. In this way, for example, novel surgical methods or implant designs can be examined early in development before clinical application. On one hand, this can save time in development, but it also minimizes the risk for patients in subsequent clinical studies [16].

The AMTI (Advanced Mechanical Technology, Inc., Watertown, MA, USA) VIVO™ (subsequently referred to as “VIVO”) is an advanced six degree of freedom (DOF) joint simulator. Being a simulator designed for cyclic load cases, wear tests can be conducted while varying intraoperative parameters like implant alignment [4]. Further applications in the area of tribology are shown by Ibrahim et al. [5] and Hossain et al. [6], investigating triboelectric energy harvesting in instrumented total knee replacements (TKRs). Fitzpatrick et al. [7] used the VIVO to validate a finite element model of a TKR that is intended to be used for wear analysis.

The VIVO simulator provides a virtual ligament model that allows more detailed modeling of joints for different experimental setups. This virtual ligament model was utilized by Sekeitto et al. [8], examining the influence of the tibial component thickness on knee joint kinematics and laxity. In this context, ligament balancing during TKR can also be examined using the virtual ligament model [9]. Furthermore, the VIVO simulator allows the investigation of different implant designs as shown by Willing et al. [10], who compared condylar-stabilized and cruciate-retaining TKRs. Benham et al. [11] added a patellar implant component to the VIVO test setup to evaluate not only tibiofemoral but also patellofemoral dynamics.

In addition to joint endoprostheses, human specimens could also be examined with the VIVO simulator. Moslemian et al. [12] investigated the biomechanical contributions of the deep medial collateral ligament and posterior oblique ligament by sequentially resecting respective ligaments and analyzing joint stability with the VIVO simulator. Andreassen et al. [13] used the VIVO simulator to validate the accuracy of a custom device for quantifying anterior–posterior (AP) and internal–external (IE) knee laxity. To investigate and assess new surgical procedures, Sidhu et al. [14] utilized the VIVO simulator to study the outcome of a surgical approach for TKR.

**Figure 1:**
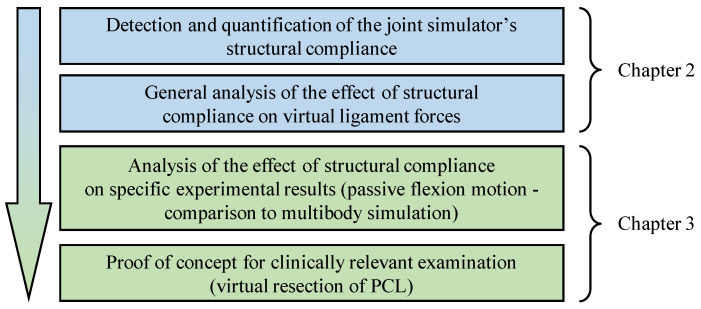
Flowchart of paper’s structure.

This paper is subdivided into two sections (see Figure 1). The objective of the first section (Chapter 2) is to demonstrate that unintended elastic deformations of the actuators due to the specifically high contact forces in the artificial knee joint exist and have a considerable impact on the ligament forces calculated by the virtual ligament model. The second section (Chapter 3) aims to demonstrate a procedure to mitigate the effects of structural compliance during the preparation and conduction of tests on a TKR. A musculoskeletal multibody system (MBS) model of an artificial knee joint developed in earlier investigations [3,15] serves as a reference for tests on the VIVO. Specific boundary conditions, e.g., positioning of implants in the bone stock and parametrization of the surrounding capsules and ligaments, are transferred to the VIVO. Experimental results are compared to numerical results obtained in the MBS. Furthermore, an exemplary clinically relevant load case virtually resecting the posterior cruciate ligament (PCL) is considered and analyzed regarding structural compliance of the joint simulator.

## 2. Analysis of the Structural Compliance of the VIVO Joint Simulator

First, the structural compliance of the VIVO’s actuators was analyzed experimentally, and the influence of elasticity is discussed.

### 2.1. Material and Methods for Structural Compliance Analysis

**Figure 2:**
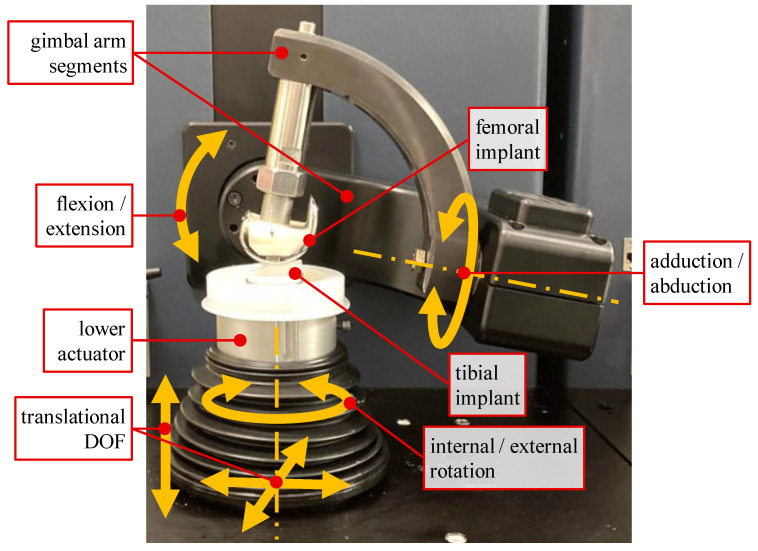
VIVO joint simulator with mounted femoral and tibial knee implants and schematic depiction of DOF. Upper actuator with two rotational DOFs for flexion/extension and adduction/abduction; lower actuator providing omnidirectional translations and internal/external rotation.

The AMTI VIVO joint simulator allows performance analyses of different types of joint implants under physiological loads. It features two servo-hydraulically driven actuators that allow for a relative motion between two articulating implant components with up to six DOF (Figure 2). The upper actuator consists of two gimbal arm segments, while the lower actuator enables medial–lateral, anterior–posterior, and distal–proximal translations as well as internal–external rotation. Each DOF can be operated in position or force/torque control mode individually. Forces and torques acting during an experiment are measured by means of a six-axis force-torque sensor, which is integrated into the lower actuator. Displacements can be measured with a resolution of 0.1 mm and 0.1°, respectively [17].

The simulator can be controlled either with a PI controller or alternatively, by means of an iterative learning control algorithm provided by the manufacturer. When using iterative learning control, a new compensation profile is generated every third load cycle to reduce tracking errors. To achieve a satisfactory reduction in errors, a sufficient number of load cycles must be chosen [18].

The influence of ligaments on joint dynamics can be considered by means of a virtual ligament model. It allows the definition of up to 100 individual virtual ligament fibers, each modeled as a non-linear elastic force element. Virtual ligament forces act on straight lines between insertion points that have constant positions in the coordinate systems of the upper- and lower-end effector, respectively. Ligament wrapping around bones or implants was not considered, as it cannot be modeled by means of the virtual ligament model. Ligament forces are calculated applying a non-linear force law introduced by Wismans [19], which exhibits quadratic behavior at low ligament strain and linear behavior at increasing strain (transition from quadratic to linear behavior at 6% ligament strain). Adjustable mechanical parameters of a ligament are its insertion points, ligament stiffness, and strain at a reference ligament length. When initiating an experiment, a reference configuration of the implant components is manually set before the virtual ligaments are activated, and the ligament insertion point locations are defined with respect to this reference configuration. During the test, the position-controlled DOFs follow predefined time functions corresponding to rheonomic constraints, while the force-controlled DOFs are moved in such a way that the static equilibrium between actual implant contact forces and virtual ligament forces is continuously established.

As the ligament forces are calculated as functions of the spatial distances between the corresponding insertion points located on the articulating implant components, exact measurement of these distances is important to take the correct ligament forces into account during testing. The insertion point locations associated with the implant components are calculated in dependence on the directly measured joint coordinates of the lower and upper actuator, respectively. From this, it follows that possible structural compliance of the actuators due to the contact force between the articulating implant components directly affects the calculation of the distances between ligament insertion points and thus of the virtual ligament forces. This applies in particular to the upper actuator with possible elastic deformations of its bearings as well as its bending- and torsion-loaded arm segments. 

**Figure 3:**
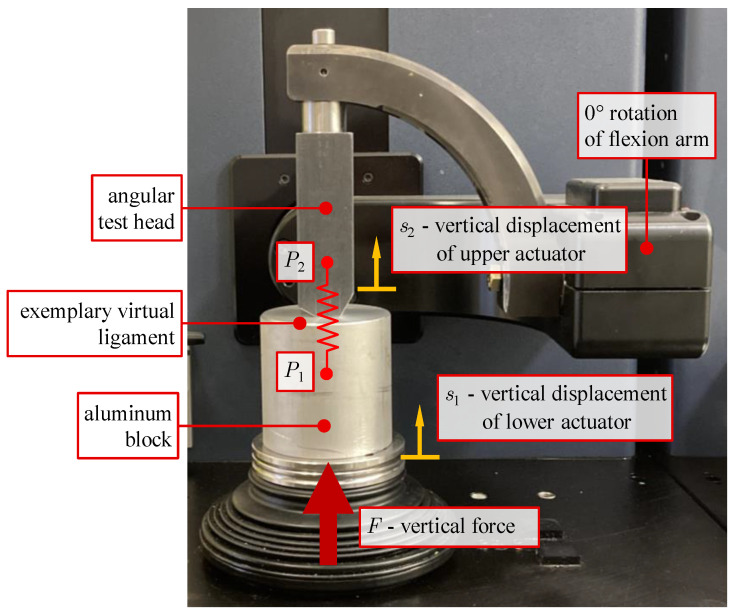
Test setup for examination of structural compliance of the VIVO at the reference configuration with 0° rotation of the flexion arm. Tests were also conducted at 30°, 60°, and 90° flexion angles.

To estimate the influence of elastic compliance, we built up the test setup shown in Figure 3. To the upper actuator, an angular test head made of steel was attached that has flat contact surfaces at the flexion angles 0°, 30°, 60°, and 90°. As a counter face, an aluminum block was mounted on the lower actuator. The underlying consideration is that the compliance of the contact between these compact metallic components is small compared to the possible compliance of the actuators. A vertical force *F* was applied for different rotations of the flexion arm, continuously increasing up to 3000 N. This force lies well within the operating range of the VIVO (maximum vertical force: 4500 N) and is a realistic force level acting in knee joints [20].

For the configuration at 0° rotation of the flexion arm, a single exemplary virtual ligament was considered (red spring in Figure 3). Ligament parametrization was adopted from a ligament (a single bundle of lateral collateral ligament) featured in the knee model that was used in subsequent investigations. The parameters according to [19] were stiffness 3300 Nperunit strain; 11% ligament strain at start of experiment (reference strain); transition from quadratic to linear force behavior at 6% strain. The ligament insertion points *P*_1_ and *P*_2_ were located on a vertical line between the angular test head and the aluminum block so that the ligament elongation was equal to the difference between vertical displacements of these points.

To verify the assumption of neglectable elastic deformations of the test components, the vertical displacement *s*_1_ measured by the VIVO simulator was compared to the vertical displacement *s*_2_ of a punch point located at the bottom end of the angular test head under the applied force *F*. The punch point displacement was measured by means of a coordinate measuring device (MicroScribe 3D, Immersion Corporation, Aventura, FL, USA). After zeroing the force–torque sensor, tests commenced with *F* = 30 N to establish initial contact.

### 2.2. Results of Structural Compliance Analysis

We could show that the displacements *s*_2_ of the upper actuator measured with the coordinate measuring device are virtually identical to *s*_1_ with a maximum deviation of 0.15 mm. They are therefore not shown separately. This result confirms the assumption of neglectable deformations of the massive test components. Therefore, the following evaluation of the actuator compliance is based on the vertical displacement of the lower actuator *s*_1_.

**Figure 4:**
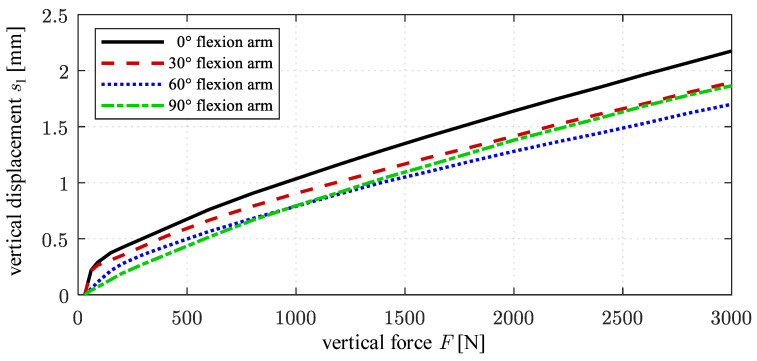
Results of structural compliance examination—vertical displacements of the lower actuator *s*_1_ over the vertical force *F* for different flexion arm rotations.

Figure 4 shows *s*_1_ over the vertical load *F* for the different angular positions of the test head. For lower loads up to approx. F = 500 N, the curves *s*_1_(*F*) degressively increase, becoming more and more linear beyond that load. Among the considered flexion arm angles, the compliance is maximal at 0° flexion arm rotation with *s*_1_ = 0.7 mm at *F* = 500 N and a subsequent gradient of approx. 0.6 mm/1000 N. It can be seen that a displacement by up to approx. 2 mm at 3000 N occurs that is caused by the compliance of the upper actuator.

**Figure 5:**
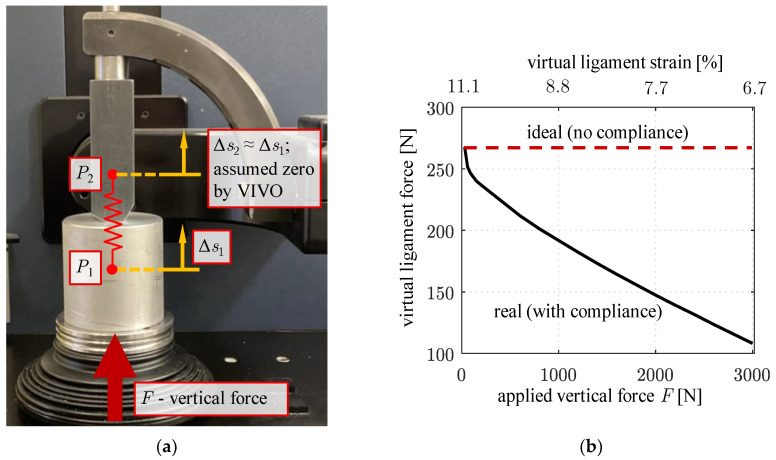
(**a**) Vertical displacements Δ*s*_1_ and Δ*s*_2_ of VIVO actuators and virtual ligament insertion points under vertical load. The elastic displacement Δ*s*_2_ is not captured by the VIVO’s sensors and is instead assumed zero for ligament force calculation. (**b**) Decreasing ligament force of the exemplary ligament under increasing vertical force. Supposing a perfectly rigid upper actuator, neither actuator would move vertically (Δ*s*_1_ = Δ*s*_2_ = 0) and the curve would be constant at the starting value of 268 N.

The elastic displacement of the upper actuator has a considerable impact on the ligament forces calculated internally in the VIVO simulator. Figure 5a schematically shows this for the exemplary ligament considered at 0° flexion arm rotation. As the angle sensors of the upper actuator do not capture the actual elastic displacement of point *P*_2_, the ligament force is calculated from the distance between the undeformed location of *P*_2_ and the actual location of *P*_1_ on the lower actuator that is known from the measured displacement *s*_1_. The virtual ligament is therefore shortened by the elastic deformation of the upper actuator under the vertical force.

This directly affected (here: decreased) the virtual ligament force with increasing contact force *F*, as can be seen in Figure 5b. As the internally calculated ligament force cannot be exported from the VIVO, it was separately calculated from the recorded vertical displacement *s*_1_ using the underlying force law. The ligament force of the exemplary virtual ligament starts at 268 N as the ligament is assumed to be pretensioned in this configuration. With increasing vertical load *F* and, by this, vertical displacement of the lower actuator *s*_1_ caused by the mechanical compliance, the calculated ligament force decreases, falling to 108 N (−60%) at the maximum vertical displacement of *s*_1_ = 2.2 mm. The impact of the mechanical compliance of the VIVO simulator on the test results using the ligament force model implemented in the VIVO is therefore significant. It becomes clear that depending on the parametrization of a ligament, a difference in the insertion point distance of less than one millimeter can considerably in- or decrease the ligament force as a function of ligament strain (see Figure 5b). Multiple ligaments in parallel connection cause inaccuracies to add up. It also follows from Figure 5b that the highest structural compliance exists at 0° flexion arm rotation, at which the highest loads simultaneously arise in the load case examined in Section 3.

The negative impact of elastic deformations on the calculated virtual ligament forces could be theoretically compensated, for example, by considering an elasto-kinematical model of the actuators of the VIVO during the calculation of ligament forces or with additional measurements of deformations. However, these solutions are excluded, as the user cannot alter the implemented ligament force calculation algorithm. An approximate compensation can be achieved by applying an increased contact force while the reference configuration before the start of a test is set. In this way, the cross table displaces in the direction of the compliance before the virtual ligament model insertion points are defined; the gimbal articulated arms are “preloaded”. A reasonable choice of this force is the average value between the expected minimum and maximum forces occurring during the load case under consideration. By this measure, the difference between the contact force in the reference configuration for which the virtual ligament insertion points are defined, and the actual force acting during a test is decreased, thus also reducing the effect of the elastic displacement on the calculated ligament forces.

## 3. Testing of a Total Knee Endoprosthesis on the VIVO Joint Simulator

For further experiments, a bicondylar PCL-retaining fixed-bearing TKR (Multigen Plus Knee System, Size 3, Lima Corporate, Udine, Italy) was examined on the VIVO joint simulator. The test setup is shown in Figure 2.

### 3.1. Material and Methods for TKR Testing on the VIVO

**Figure 6:**
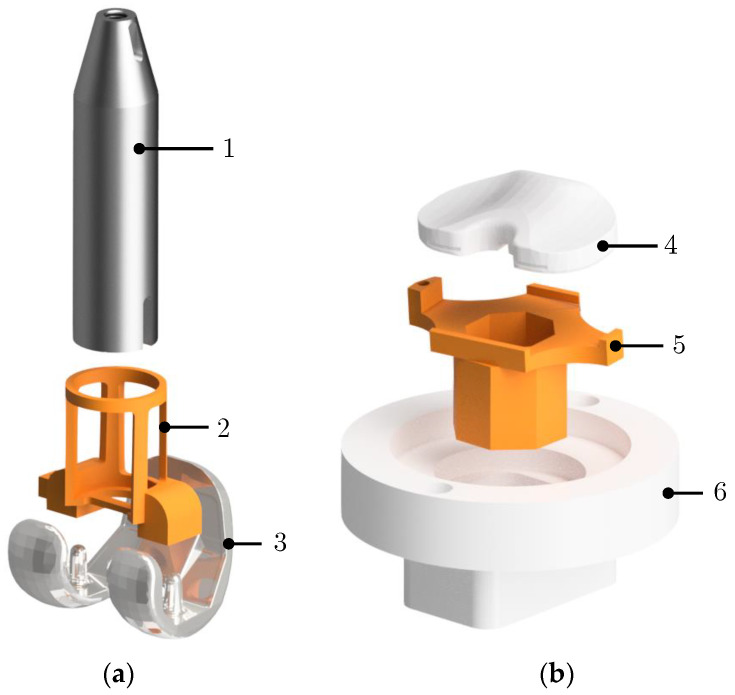
Functionality of embedding templates. Shown parts are merged with epoxy resin: (**a**) femoral component: holder 1, embedding template 2, implant 3; (**b**) tibial component: tibia insert 4, embedding template 5, holder 6. Holders 1 and 6 feature coupling interfaces matching the VIVO’s actuators.

The implants were mounted onto the actuators of the simulator using mechanical holders. To achieve a defined positioning of the implants, 3D-printed embedding templates (printed with Stratasys Objet Connex500, Stratasys Ltd., Eden Prairie, MN, USA) were used, which provide a form-fitting connection between implants and respective holders (Figure 6). Among other requirements, the embedding templates must ensure that the femoral implant’s flexion axis coincides with the VIVO simulator’s gimbal flexion axis. In addition, the vertical tibial implant axis must be orthogonal to the plane of the mounting plate of the lower actuator [17]. Subsequently, the implants were molded in epoxy resin, which provided a rigid connection to the holders. To verify the positioning of the implants after embedment, a geometric measuring method was applied using a coordinate measuring device [15]. After measuring the coordinates of distinct geometrical landmarks on both implants, the respective implant-fixed coordinate systems could be reconstructed. The coordinates of each measuring point were taken three times individually, with the average of these values being used. Minor corrections of embedding inaccuracies on translations and internal-external-rotation of the tibia insert, as well as rotations of the femoral implant about its flexion/extension axis, could be achieved by moving the respective actuators before defining the reference configuration.

The following virtual ligaments were implemented by means of the virtual ligament modeling options of the VIVO simulator: two bundles of PCL; three bundles of medial and lateral collateral ligament (MCL/LCL), respectively; one bundle of oblique posterior medial collateral ligament (opMCL); two bundles of deep medial collateral ligament (dMCL); two bundles of oblique popliteal ligament (OPL); one bundle of arcuate popliteal ligament (APL); and one bundle of medial and lateral posterior capsule (mpCAP, lpCAP), respectively. Mechanical properties (stiffness, reference strain) and insertion point locations of ligaments were adopted from [3,21]. A patellar component was not considered in this study.

**Figure 7:**
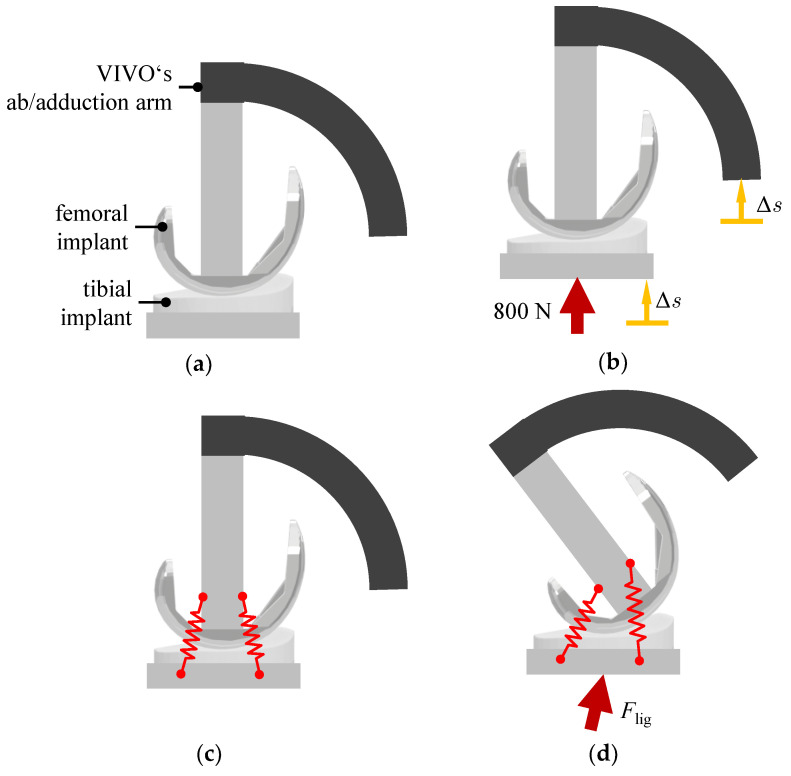
Setting of reference configuration and definition of virtual ligaments: (**a**) The relative position between implants obtained in the MBS is set kinematically; (**b**) a given initial vertical force is applied, causing both actuators to move up vertically by Δ*s*. The resulting configuration is defined as reference configuration. (**c**) The virtual ligament insertion points are defined in the reference configuration. Virtual ligaments are visualized as springs. (**d**) Passive flexion motion is executed, during which the resulting force of all individual virtual ligament forces *F*_lig_ is applied by the lower actuator.

To evaluate the VIVO experiments and findings regarding limitations, we simulated an exemplary load case in the form of a passive flexion motion from 0° to 80°. Before conducting the experiments, the aforementioned reference configuration between implants was set in order to define the virtual ligament insertion point locations. The reference configuration is the equilibrium configuration of the knee joint under the influence of ligaments at 0° flexion. It was obtained from a musculoskeletal MBS developed in earlier investigations [3,15]. After this relative position between implants was set kinematically (Figure 7a), a given initial vertical contact force was applied for approximate compensation of elastic deformations as described in Section 2.2. The chosen force magnitude, in this case 800 N, is the average value between the expected minimum and maximum forces occurring during the load case. This caused both actuators and implants to displace further vertically by Δ*s* due to the structural elasticity of the VIVO (Figure 7b). The resulting actuator configuration was set as the reference configuration. Next, the virtual ligament insertion points are defined in the reference configuration. Virtual ligaments (visualized as springs) run between respective insertion points (Figure 7c). Finally, a passive flexion motion is executed, during which the virtual ligament insertion points are moving together with their respective implants. Calculated individual ligament forces act between insertion points. The resulting force of all individual virtual ligaments *F*_lig_ depending on ligament parametrization and actuator positions is applied by the lower actuator (Figure 7d).

Tests were conducted using silicone oil (viscosity 360 mm^2^s^−1^) as lubricant. The VIVO’s iterative learning control algorithm was applied, whereby 300 load cycles were conducted for each specific test. It turned out that more than 300 load cycles do not further decrease root mean square errors of control deviations for each force- or position-controlled DOF, showing that the iterative learning control algorithm has reached its maximum control accuracy for this specific load case. Averages of the final three load cycles of each test were evaluated. To rule out the influence of the flexible bellows, which are intended to protect the force–torque sensor from lubricant and impurities, on measured joint forces and torques, bellows were removed for all tests [18].

As a parameter study, a gradual resection of the PCL was simulated. The investigation of the influence of PCL resection on joint dynamics has been the subject of previous studies [21,22,23,24,25] and shall demonstrate the viability of the VIVO joint simulator for analyzing clinically relevant questions. Virtual PCL resection is achieved by incrementally reducing the stiffness value of both modeled PCL bundles. The remaining test parameters (load case, parametrization of other ligaments, contact force at setting of reference position, lubrication, number of load cycles) remain unchanged.

**Figure 8:**
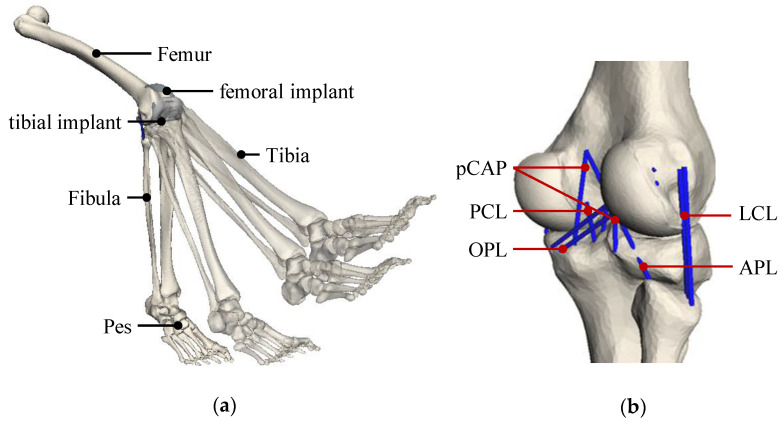
Musculoskeletal multibody model: (**a**) open kinematic chain with bones and implants shown at different flexion angles. (**b**) Detailed view of the knee joint model (posterior–lateral view) with considered ligament bundles (MCL not visible, implants hidden for clarity). These ligaments were also considered in the VIVO experiments.

Results obtained with the VIVO are compared to results of a musculoskeletal MBS simulation of the considered tibiofemoral joint with the bicondylar TKR. The MBS model (Figure 8) was built up in SIMPACK 9.7 (Dassault Systèmes, Vélizy-Villacoublay, France) and consists of 3D surface geometries of bony structures of the lower extremity and the implant components. The ligaments were modeled exactly as in the VIVO experimental setup. Accordingly, ligament wrapping was not considered. The MBS was modeled as an open kinematic chain, with the femur being fixed and the tibia/fibula and pes being moveable. A polygonal contact model [26] was defined between the articulating surfaces of the implant components. To simulate the passive flexion according to the VIVO experiment, the tibiofemoral joint had five DOF, while the flexion angle was predefined as a rheonomic constraint. The simulation provides the remaining joint translations and rotations of the tibia from the equilibrium conditions between joint contact and ligament forces. The MBS knee model was developed, applied, and validated in preceding investigations [15,21].

### 3.2. Results of TKR Testing on the VIVO

#### 3.2.1. Passive Flexion—Comparison with Multibody Simulation

**Figure 9:**
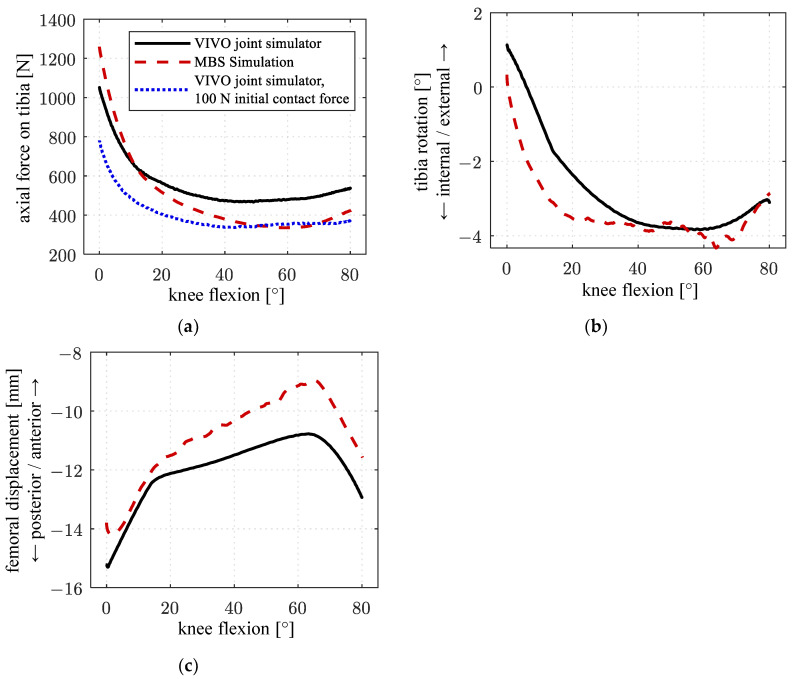
Results of passive flexion test on the VIVO in comparison to MBS simulation: (**a**) axial contact force on tibia; (**b**) tibia internal/external rotation; (**c**) femoral AP displacement. Anatomical directions are indicated by arrows on the vertical axis.

With the test setup (Figure 1), passive flexion of the artificial knee joint was simulated on the VIVO and compared with the MBS simulation. Figure 9a compares the results for the absolute value of the contact force acting on the tibia in the direction of the mechanical tibial axis. The result of a comparative simulation in which the vertical force in the reference configuration was only 100 N instead of 800 N again shows the influence of the actuator compliance that reduces the ligament force level as already seen in Figure 5b. Figure 9b,c shows the corresponding tibiofemoral kinematics under consideration of tibial rotation as well as femoral AP displacement relative to the tibia. Kinematic quantities are represented with respect to the reference configuration at 0° flexion.

Similarities in shape can be noted for all curves of the characteristics considered. Between the MBS simulation and the VIVO experiment conducted after obtaining a reference pose with 800 N contact force (black/solid and red/dashed), forces generated in the VIVO experiment are lower at low flexion angles (−246 N/−19% at 0° flexion) but higher at increasing flexion (approx. +120 N or +35% between 50° and 80° flexion). When comparing axial forces on the tibia in both VIVO simulator experiments (black/solid and blue/dotted), the force level is considerably lower (overall approx. 25%, maximal deviation of −250 N at 0° flexion) in the test conducted after obtaining the reference configuration with a force of 100 N. The highest deviation between all curves occurs when comparing the MBS simulation to the experiment conducted with a 100 N force at the reference configuration (red/dashed and blue/dotted), with the force generated by the VIVO simulator being 500 N/60% below the force in the MBS simulation at 0° flexion. However, it must be noted that the corresponding curves converge at increasing flexion angles. Considering the rotation of the tibia, terminal rotation occurs over a larger flexion range in the VIVO experiment (0° to 40° flexion) in comparison to the MBS simulation (0° to 20° flexion). The overall femoral displacement is more posterior in the VIVO experiment with a maximum deviation of approx. 2 mm (−22%) at the point of maximal anterior femoral displacement (65° flexion for both curves).

#### 3.2.2. Passive Flexion—Virtual Resection of PCL

**Figure 10:**
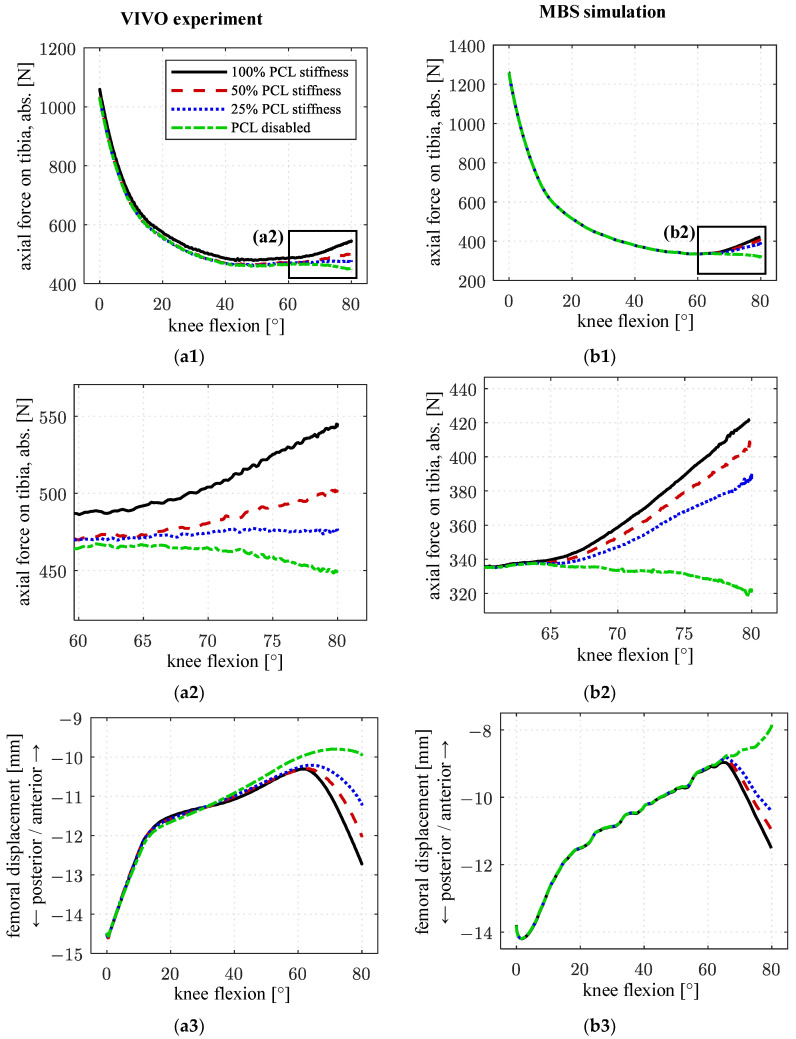
Results for different PCL stiffnesses during passive flexion. (**a1**–**a3**): VIVO experiment; (**b1**–**b3**): MBS simulation; (**a1**,**b1**) axial contact force on tibia; (**a2**,**b2**) detail view of (**a1**,**b1**) at high flexion angles; (**a3**,**b3**) femoral AP displacement.

Simulating the same load case (passive flexion), the axial tibia force as well as the femoral AP displacement were evaluated for 100%, 50%, 25%, and 0% PCL stiffness, respectively. The results are shown for the VIVO experiment (Figure 10(a1–a3)) as well as the MBS simulation (Figure 10(b1–b3)).

During low flexion angles, all curves showed a similar shape. Starting at approx. 65° of flexion, the axial force curves diverge with the force absolute decreasing with reduced PCL stiffness. In the VIVO experiment, the force difference between 100% PCL stiffness and a disabled PCL was approx. 90 N at 80° of flexion. In the MBS simulation, a force difference of 100 N occurred at 80° of flexion, with the overall force level at high flexion angles being lower than in the VIVO experiment for all PCL stiffnesses as already observed in the preceding experiment (Figure 9a).

Considering femoral AP displacement, starting at approx. 60° of flexion, a decrease in femoral rollback could be observed, which is more prevalent with successive PCL stiffness reductions for both the experiment and the simulation. With the PCL disabled, femoral posterior motion was almost nonexistent in the VIVO experiment, while the femur moved exclusively anteriorly in the MBS simulation. The maximum femoral AP displacement difference between 100% PCL stiffness and the disabled PCL was approx. 2.8 mm at 80° of flexion in the VIVO experiment, and approx. 3.6 mm at 80° of flexion in the MBS simulation.

## 4. Discussion

### 4.1. Passive Flexion—Comparison of VIVO Experiment with Multibody Simulation (Figure 9)

All data generated by the VIVO simulator show typical characteristics for passive knee flexion, which were also obtained in the reference multibody simulation. Notable are the increasing axial joint force, as well as the tibial terminal rotation (screw-home mechanism) at low flexion angles, and the femoral rollback at increasing knee flexion, starting at approx. 65° [1,21,22].

At low flexion angles (high axial force level), axial forces generated by the VIVO simulator are below the corresponding results from the multibody simulation. In contrast, with an increasing flexion angle and decreasing axial force levels, forces generated by the VIVO simulator exceed those calculated in the multibody simulation. This again shows the effect of the structural compliance of the VIVO actuator in connection with the reference configuration contact force of 800 N. Exceeding this value causes increased displacement of the cross table in the proximal direction, shortening virtual ligaments and decreasing ligament forces. This effect reverses if the joint force falls below the reference configuration contact force, increasing insertion point distances and resulting in higher ligament forces. The effect is further proven when comparing only the curves obtained in the VIVO experiments with different reference configuration forces (black/blue in Figure 9a): An overall, considerably decreased force level can be observed for the experiment with less reference configuration force, while the curve shapes are virtually identical. This proves that preloading the simulator with a median force when setting the reference configuration can decrease but not avoid the influence of structural compliance on virtual ligament forces.

An overall more posterior position of the femur relative to the tibia during flexion suggests reduced sliding motion between the femur and tibia, while posterior translation caused by rolling of the femoral condyles on the tibia plateau during flexion is more predominant. This indicates increased friction between the contact partners in the experimental simulator setup compared to the numerical MBS simulation. By adjusting (increasing) the friction coefficient between the implant components in the MBS, femoral AP translation in the MBS simulation became more consistent with the VIVO experimental setup, proving this assumption.

### 4.2. Passive Flexion—Virtual Resection of PCL (Figure 10)

Decreasing the PCL stiffness has an influence on the joint dynamics of the artificial knee joint. Consecutively, at high flexion angles, femoral rollback and tibiofemoral contact force are reduced. No differences can be observed at flexion angles below 60°. The results obtained in the VIVO experiment exhibit similar characteristics to the MBS simulation. These characteristics are expected, as the PCL gets more tensioned with increased flexion and is responsible for femoral rollback [1,27]. The results are also in accordance with findings from preceding investigations [21,28,29]. Despite the previously described findings regarding structural compliances and their effect on virtual ligament forces, this shows the capability of the VIVO joint simulator to investigate clinically relevant topics by using a virtual ligament model. Similar characteristics between forces and kinematics derived from the VIVO experiment and MBS simulation prove that data generated in previous examinations can be reproduced under varying boundary conditions.

## 5. Limitations of the Present Study

Some limitations were identified in our present study. The geometric measuring method applied to verify the position of the implant components was conducted using a coordinate measuring device. For this, it was required to manually position the measuring probe as precisely as possible at predefined points, during which minor deviations cannot be ruled out. Furthermore, only one load case (passive flexion motion) and one TKR design were examined. While other load cases like the squat or gait are considered more relevant in terms of clinical examinations [30,31], the passive flexion was chosen as a basic load case that could be compared to similar previous investigations [21] to verify presented methods and assumptions. The TKR used in this study was chosen because it was the subject of preceding investigations [15,21], which served as a reference for this study. The methodology shown can also be applied to other implant designs (e.g., posterior–stabilized or mobile bearing). When comparing the findings of the present study to results from the literature, it must be considered that the experimental results are affected by the structural compliance of the VIVO simulator as described. In addition, the absence of a patellar implant component affects joint dynamics as well and can represent an important factor in TKR dynamics [11,32]. The consideration of ligament wrapping also influences the dynamics of the artificial knee joint, as it helps to ensure that non-physiological motions do not occur [33], but consideration of wrapping is not feasible by means of the virtual ligament model. Another limitation is the use of silicone oil as a lubricant. This has been established in the literature for the dynamic and kinematic testing of TKR [8,18], though it does not have the same rheological properties as human synovial fluid [34]. In the future, studies with different samples of the highly variable human synovial fluid [35] should therefore be conducted to analyze possible effects on joint kinematics and dynamics.

## 6. Conclusions

In the present study, a method to prepare and conduct tests on TKR by means of the VIVO joint simulator was introduced. Data obtained in a musculoskeletal multibody simulation were reproduced in experiments using the VIVO simulator. Limitations of the joint simulator caused by the elastic compliance of the actuators under load were identified. This especially affects the calculation of virtual ligament forces by means of the model implemented in the simulator. When conducting tests for which the exact acting of joint contact forces is crucial (e.g., wear tests), we recommend refraining from using the virtual ligament model and applying predefined force curves instead. A possible measure to reduce the influence of structural compliance by preloading the actuators before the experimental testing was presented. Further compensation for compliance may be achieved by incorporating an elasto-kinematical model of the actuator system into the calculation of ligament forces or by directly measuring elastic deformations.

The functionality and advantages of the VIVO simulator and especially the consideration of the ligament model could be proven in an exemplary study, virtually decreasing the stiffness of the PCL. In future research, a patellar implant shall be physically implemented, which can be loaded by an actuator representing the quadriceps muscles.

## Data Availability

The original contributions presented in this study are included in the article; further inquiries can be directed to the corresponding author.
